# Development, functional organization, and evolution of vertebrate axial motor circuits

**DOI:** 10.1186/s13064-018-0108-7

**Published:** 2018-06-01

**Authors:** Kristen P. D’Elia, Jeremy S. Dasen

**Affiliations:** Neuroscience Institute, Department of Neuroscience and Physiology, NYU School of Medicine, New York, NY 10016 USA

**Keywords:** Motor neuron, Spinal circuit, Neural circuit, Development, Evolution, Axial muscle

## Abstract

Neuronal control of muscles associated with the central body axis is an ancient and essential function of the nervous systems of most animal species. Throughout the course of vertebrate evolution, motor circuits dedicated to control of axial muscle have undergone significant changes in their roles within the motor system. In most fish species, axial circuits are critical for coordinating muscle activation sequences essential for locomotion and play important roles in postural correction. In tetrapods, axial circuits have evolved unique functions essential to terrestrial life, including maintaining spinal alignment and breathing. Despite the diverse roles of axial neural circuits in motor behaviors, the genetic programs underlying their assembly are poorly understood. In this review, we describe recent studies that have shed light on the development of axial motor circuits and compare and contrast the strategies used to wire these neural networks in aquatic and terrestrial vertebrate species.

## Background

The neuromuscular system of axial skeleton plays crucial roles in basic motor functions essential to vertebrates, including locomotion, breathing, posture and balance. While significant progress has been made in deciphering the wiring and function of neural circuits governing limb control [1, 2], the neural circuits associated with axial muscles have been relatively under studied, particularly in mammals. Despite comprising more than half of all skeletal muscles in mammals, how axial neural circuits are assembled during development is poorly understood.

Although all vertebrates share similar types of axial muscle [3, 4], the nervous systems of aquatic and terrestrial species control these muscle groups in distinct ways. In most aquatic vertebrates, rhythmic contraction of axial muscle is essential for generating propulsive force during swimming, the predominant form of locomotion used by fish. In land vertebrates, axial circuits have been largely dissociated from locomotor functions, and have been modified throughout evolution to enable new types of motor capabilities. In animals with upright postures, neuronal control of axial muscles is essential to maintain balance and proper alignment of the spine. During the invasion of land by vertebrates, axial muscles that were initially used in swimming were also adapted by the respiratory system to enable breathing in air. Since many of these diverse axial muscle-driven motor behaviors are encoded by neural circuits assembled during development, insights into the evolution of axial circuits might emerge through comparisons of the genetic programs that control neural circuit assembly in different animal species.

In this review, we discuss studies which have investigated the development, evolution, and wiring of neuronal circuits essential for control of axial muscle. Recent advances in genetically tractable systems, such as zebrafish and mouse, have provided novel insights into the mechanisms through which axial circuits are assembled during development, and have shed light on the wiring of the circuits essential for balance, breathing, and locomotion. We compare the strategies through which animals generate distinct classes of spinal neurons that coordinate axial muscles, with particular focus on the spinal motor neuron subtypes that facilitate axial-driven motor behaviors.

## Functional organization and peripheral connectivity of axial motor neurons

**Fig. 1:**
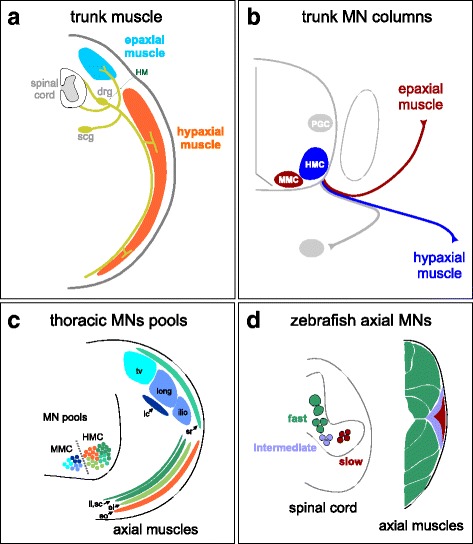
Organization of axial MNs in tetrapods and fish. **a** In jawed vertebrates, axial muscles are separated into dorsal epaxial and ventral hypaxial groups, separated by the horizontal myoseptum (HM). Each muscle group is innervated by separate spinal nerves. Dorsal root ganglia (drg) and sympathetic chain ganglia (scg) are shown. **b** MN columnar subtypes at trunk levels. In tetrapods, as well as some cartilaginous fish, MNs innervating dorsal epaxial muscles are organized in the medial motor column (MMC). MNs projecting to ventral hypaxial muscles are contained within the hypaxial motor column (HMC). Autonomic preganglionic column (PGC) neurons, which project to scg, are shown in gray. **c** Organization of MN pools at thoracic levels. MNs innervating specific types of axial muscle are organized in pool-like clusters. Some MNs within the HMC project to dorsally located axial muscles, such as serratus, but are nevertheless supplied by axons originating from the ventral ramus. Abbreviations: tv, transversospinalis; long, longissimus; ilio, iliocostalis; lc, levator costae; sr, caudal serratus; ii, internal intercostal; sc, subcostalis; ei, external intercostal; eo, external oblique. Not all trunk muscles are shown. Diagram based on data from rat in [13]. **d** Organization of MNs in adult zebrafish. MNs innervating fast, intermediate, and slow muscle are organized along the dorsoventral axis. Fast MNs include primary MNs and some secondary MNs, intermediate and slow are all secondary MNs. These MN types project to specific types of trunk-level axial muscles. Diagram based on data in [14]

Although used for fundamentally distinct motor functions, the axial neuromuscular systems of fish and tetrapods share many anatomic features and early developmental programs [3, 4]. In both fish and tetrapods, axial muscles can be broadly divided into two groups, epaxial and hypaxial, which are initially separated by a horizontal myoseptum (Fig. 1a). Epaxial muscles reside dorsal to the myoseptum and include muscle groups associated with the vertebral column and base of the skull. Hypaxial muscles are predominantly located ventral to the mysoseptum and give rise to diverse muscle groups including abdominal and intercostal muscles, as well as the diaphragm in mammals. In tetrapods, migratory populations of hypaxial muscle also generate all of the muscle in the limb. In fish and amphibians, the separation between dorsal and ventral axial muscles is maintained in adulthood, while in tetrapods many of these positional differences have been lost. Both types of axial muscles receive innervation from spinal motor neurons (MNs) and sensory neurons that project either along the dorsal (epaxial) or ventral (hypaxial) branches of the spinal nerves.

In tetrapods, MNs targeting specific muscle groups are organized in discrete clusters, termed motor columns and motor pools [5–8]. Spinal MNs projecting to functionally related muscle groups, such as epaxial, hypaxial, or limb muscle, are contained within motor columns that occupy specific rostrocaudal positions within the spinal cord. Within these columnar groups, MNs further segregate into motor pools, each pool targeting a single muscle. Each pool occupies a specific position within the spinal cord, and its relative position along the dorsoventral, mediolateral, and rostrocaudal axes is linked to how MNs project within a target region. The stereotypic organization of MN position within the spinal cord therefore establishes a central topographic map which relates neuronal settling position to target specificity.

Studies on the developmental mechanisms controlling MN columnar and pool organization have largely focused on the diverse subtypes innervating limb muscles [9, 10]. Axial MNs also display a topographic organization that relates neuronal position to target specificity. The cell bodies of MNs targeting epaxial and hypaxial muscles are organized in specific columnar groups within the ventral spinal cord (Fig. 1b). Dorsal epaxial muscles are innervated by MNs in the median motor column (MMC), while hypaxial muscles are innervated by MNs in the hypaxial motor column (HMC). MMC neurons occupy the most medial position of all spinal MNs, whereas HMC neurons, and all other MN subtypes, typically reside more laterally [11]. Like limb MNs, both MMC and HMC neurons further differentiate into specific pool groups, and axial MN pool position is linked to the location of its muscle target (Fig. 1c). For example, MMC neurons targeting more dorsal epaxial muscles reside more medially than those targeting more ventral muscle [12]. A similar somatotopic organization has been observed for HMC pools targeting different intercostal and abdominal muscles [13].

In contrast to tetrapods, the organization of axial MNs into well-defined columnar groups has not been described in zebrafish. Despite the absence of an obvious columnar organization, zebrafish axial MNs are functionally organized along the dorsoventral axis of the spinal cord (Fig. 1d). This organization is associated with how MNs are recruited at distinct swimming speeds and correlated with the type of muscle a MN innervates, as opposed to the location of the muscle. Axial MNs projecting to muscles activated at slow swimming speeds reside ventrally, MNs recruited at fast swimming speeds are located dorsally, and MNs involved in intermediate speeds sit between fast and slow MNs [14–16].

Although a clustered organization of axial MN has not been described in zebrafish, in certain cartilaginous fish species, including the little skate and catshark, the cell bodies of MMC neurons are clustered and settle in a ventral position [17]. These observations suggest that the organization of axial MNs into columns was present in the common ancestor to cartilaginous fish and tetrapods, and therefore to all jawed vertebrates with paired appendages. Notably, unlike most fish species, skates do not use the axial muscles to generate propulsive force during locomotion, which is instead provided by contraction of the pectoral and pelvic fins. The organization of MNs into columnar and pool groups therefore does not appear to have evolved with terrestrial locomotion, but rather reflects differences that emerged between certain fish species and other vertebrate classes.

## Genetic programs specifying early axial motor neuron fates

**Fig. 2:**
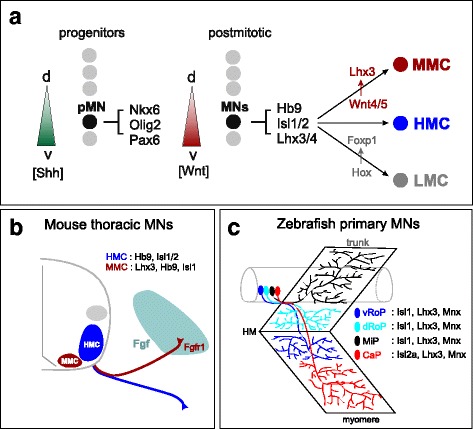
Specification of axial MNs in tetrapods and fish. **a** Specification of early axial MN identities. Graded sonic hedgehog (Shh) acts along the dorso (d)-ventral (v) axis to specify MN progenitors (pMN) and ventral interneuron fates. Graded Wnt signaling promotes sustained expression of Lhx3 in MMC neurons, while Hox signaling specifies segmentally-restricted MN columnar fates, including limb-innervating lateral motor column (LMC) neurons. **b** Axial MNs in tetrapods can be defined by expression of specific transcription factors. MMC neurons express *Fgr1* and are attracted to mesodermally-derived FGF signaling. **c** Primary MNs in zebrafish. Four distinct axial MN types can be defined by their rostrocaudal position and muscle target specificity. dRoP, dorsal rostral primary; vRoP, ventral rostral primary; CaP, caudal primary; MiP, middle primary MN

How are the distinct identities of MMC and HMC neurons established during tetrapod development? As with other subtypes of spinal MNs, the progenitors that give rise to axial MNs are specified through secreted signaling molecules acting along the dorsoventral axis of the neural tube shortly after its closure [18]. These morphogens establish specific molecular identities through the induction of transcription factors in neuronal progenitors, which subsequently specify the identity of each of the major classes of spinal neuron. In the ventral spinal cord, graded Shh signaling induces expression of transcription factors which specify MN and ventral interneuron progenitor identities [19]. As progenitors differentiate, additional transcription factors are expressed within postmitotic cells and act to define specific neuronal class fates [20]. Spinal MN progenitors are derived from a domain characterized by expression of Olig2, Nkx6.1, and Pax6. As postmitotic MNs emerge, they initially express the Lim homeodomain proteins Islet1, Islet2 (Isl1/2), Lhx3, Lhx4 (Lhx3/4), as well as the Mnx-class protein Hb9 (Fig. 2a).

As MNs differentiate and migrate to their final settling positions, subtypes of axial MNs can defined by differential expression of Lim HD and Mnx factors [11, 21]. In tetrapods, MMC neurons maintain expression of Hb9, Isl1/2, and Lhx3/4, whereas the majority of other MN subtypes, including HMC neurons, downregulate Lhx3 as they become postmitotic (Fig. 2b**)**. The specific functions of *Lhx3* and *Lhx4* in MMC neurons are not completely understood, as both genes are required for the differentiation of all spinal MN subtypes [22]. Nevertheless, misexpression of Lhx3 can convert limb MNs to an MMC fate and redirect motor axons towards axial muscle, indicating that Lhx3 plays an instructive role in determining the trajectories of MMC motor axons towards epaxial muscle [23]. While trunk-level HMC neurons can be also defined by expression of specific transcription factor combinations, whether these factors are required for columnar-specific differentiation programs is currently unknown.

A key step in the specification of axially-projecting MNs is the segregation of newly born neurons into MMC and HMC subtypes. MMC neurons are thought to represent the ancestral “groundstate” of MNs from which all other subtypes subsequently evolved [24]. This idea is supported by the observation that MMC identity is the default differentiation state of MNs derived from embryonic stem cells (ESCs) generated through induction with retinoic-acid and Shh [25, 26]. In addition, MMC-like neurons drive locomotor behaviors in limbless vertebrates such as the lamprey and insect larvae, suggesting that an MMC-like MN population represents the ancestral condition of MNs in bilaterians.

In tetrapods, an obligate step in MMC differentiation is the sustained expression of Lhx3/4 in post-mitotic MNs; while in HMC neurons and all other MN subtypes Lhx3/4 must be downregulated for proper differentiation [21, 23]. The maintenance of Lhx3/4 in MMC neurons appears to be partially governed by Wnt signaling originating from near the floorplate of the spinal cord (Fig. 2a) [27]. Overexpression of *Wnt4* or *Wnt5a* promotes the specification of MMC neurons at the expense of other MN subtypes in chick embryos, while combined genetic removal of *Wnt4*, *Wnt5a*, and *Wnt5b* in mice leads to depletion in MMC number. Recent studies in ES cell-derived MNs suggest that additional signaling pathways act in conjunction with Wnt signaling to promote MMC specification [28]. Inhibition of Notch signaling in ES-cell derived MNs promotes the specification of HMC neurons at the expense of MMC neurons, suggesting that Wnt4/5 and Notch cooperate to specify MMC identity.

While the extrinsic and intrinsic factors governing the specification of MMC and HMC neurons have been characterized, the downstream effectors of their fate determinants are less well-understood. Soon after leaving the cell cycle, the axons of MMC and HMC neurons begin to project outside the spinal cord, both initially pursuing ventrolateral trajectories. The axons of MMC neurons separate from the main nerve and extend dorsally, while all other MN subtypes, including HMC neurons, continue to extend ventrolaterally. The dorsal trajectory of MMC neurons appears to rely on target-derived chemoattractant signaling emanating from a somite-derived structure, the dermomyotome [29, 30]. This region expresses fibroblast growth factors (FGFs) which act on the axons of MMC neurons that selectively express FGF receptor 1 (Fgfr1) (Fig. 2b) [31]. Mutation of *Ffgr1* in mice causes defects in the peripheral trajectory of MMC axons. In addition, misexpression of Lhx3 leads to ectopic expression of *Fgfr1* in non-MMC MNs and causes limb motor axons to gain sensitivity to FGFs [31].

## Specification of axial MNs in zebrafish

In zebrafish, spinal MNs innervating axial muscle are specified by the same core groups of transcription factors that act in tetrapods. Unlike amniotes, where all MNs are generated during a single wave of neurogenesis, zebrafish have two waves of MN birth, primary and secondary. Primary and secondary neurons are each important for different types of axial muscle-based behaviors, but not distinguished by any known transcription factor [32, 33]. Primary MNs, which number three to four per hemi-segment, are born between 10 and 14 hours post-fertilization (hpf), develop subtype-specific electrical membrane properties as early as 17 hpf, and begin axon initiation at 17 hpf [34, 35]. Although one or two common MN markers such as Isl1, Isl2, and Mnx proteins can help differentiate two or three primary MN subtypes at different ages, these factors cannot distinguish them throughout development and have dynamic expression patterns that make the subtypes challenging to track over time [36–38]. All early-born MNs require the Olig2 transcription factor [39], while Nkx6 proteins appear to be required only in a subset of primary MNs [40]. Postmitotic primary MNs can be defined by differential expression of Mnx/Hb9, Isl1/2, and Lhx3 factors [37, 38, 41–43].

Most genetic studies of axial MN specification in zebrafish have largely focused on the specification of the four major types of primary MNs: the dorsal rostral primary (dRoP), ventral rostral primary (vRoP), caudal primary (CaP), and middle primary (MiP) subtypes (Fig. 2c). dRoP and MiP MNs are similar to MMC neurons, in that they project to muscles located dorsal to the horizontal myoseptum, while CaP and vRoP project ventrally. However, unlike MMC and HMC neurons in tetrapods, these primary MN types cannot be distinguished by differential expression of Lhx3. Nevertheless, disruption of the core MN determinants Lhx3/4, Isl1/2, and Mnx leads to defects in primary MN specification and connectivity. For example, loss of Lhx3/4 leads to MNs with hybrid MN/interneuron fates [41], while loss of Mnx proteins affects the specification of MiP MNs [38].

While much is known about primary axial MNs, the later-born secondary MNs have been particularly understudied. Although they make up the majority of spinal MNs in zebrafish, and are thought to be more similar to mammalian MNs, very little is known about their differentiation programs [44]. Secondary MNs are born starting at 16 hpf, begin axon initiation at 26 hpf, and are produced to an undetermined time after 25 hpf [35]. Multiple studies have described up to ten different axial-muscle innervating subtypes, six of those are secondary MNs [45]. All MN subtypes can be differentiated based on birthdate, muscle target, soma size and position, presence or absence of intraspinal or intermyotomal collaterals, and firing properties. There are three distinct types of firing patterns expressed by zebrafish axial MNs at 4 dpf: tonic, chattering, and burst firing. Tonic firing patterns are specific to primary MNs, while chattering and burst firing patterns are specific to secondary MNs. Each secondary MN subtype has a different distribution of these two firing patterns. While the distinct physiologic and anatomic features of secondary MNs have been well-characterized, it is yet unknown whether they reflect the operation of MN-intrinsic genetic programs acting during development.

## Diversification of tetrapod axial motor columns

While axial MNs of fish and mammals share several common early developmental programs, in tetrapods these subtypes have undergone a significant degree of modification throughout the course of vertebrate evolution. All of the segmentally restricted subtypes of spinal MNs, including the diverse MN populations innervating limb muscle, appear to have evolved from the ventrally-projecting HMC-like population. This hypothesis is supported by the observation that in genetic mutants with disrupted specification of non-axial MN subtypes, affected populations revert to a predominantly HMC-like molecular profile. Genetic deletion of the limb MN fate determinant *Foxp1* in mice causes a loss of limb-specific MN programs and an expansion in the number of MNs with an HMC-like molecular identity [21, 46]. Expression of *Foxp1* in limb-innervating lateral motor column (LMC) neurons is governed by Hox transcription factors expressed at specific rostrocaudal levels of the spinal cord, and *Hox* genes are essential for generating the diverse motor pool populations targeting specific limb muscles [47–49]. MMC neurons appear to be insensitive to the activities of Hox proteins, likely due to the functionally dominant actions of Lhx3 [21, 23]. The diversification of tetrapod spinal MNs appears to stem from HMC-like precursors which co-opted *Hox* genes to generate more specialized populations.

**Fig. 3:**
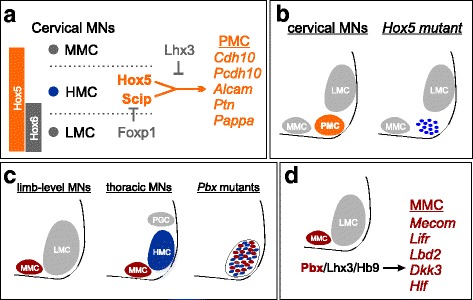
Diversification of axial MN subtypes in tetrapods. **a** At rostral cervical levels, HMC-like precursors give rise to phrenic motor column (PMC) neurons through the actions of Hoxa5 and Hoxc5 proteins. The activities of Hox5 proteins are inhibited by Lhx3 in MMC neurons, and Foxp1 in LMC neurons. Hox5 proteins work in conjunction with the Pou domain protein Scip to promote PMC-restricted gene expression. **b** In the absence of *Hox5* genes, PMC neurons are disorganized and revert to an HMC-like state. **c** *Pbx* genes are required for the columnar organization of axial MNs. In the absence of *Pbx* genes, Hox-dependent MN subtypes (LMC and PGC neurons) are lost, and acquire an HMC fate. The remaining HMC and MMC subtypes are disorganized at all spinal levels. **d** Pbx proteins act in conjunction with other MMC-restricted factors such as Lhx3 to promote MMC specific gene expression

Hox-dependent regulatory programs also contributed to the diversification of MNs targeting specific hypaxial muscle types. An important step in the evolution of mammals was the appearance of a novel MN subtype dedicated to control of respiratory muscles. MNs innervating the diaphragm are contained within the phrenic motor column (PMC) and require the actions of two *Hox* genes (*Hoxa5* and *Hoxc5*) for their specification [50]. Similar to the role of *Foxp1* in limb MNs, loss of *Hox5* genes disrupts PMC specification programs and diaphragm innervation, with the remaining MNs reverting to a thoracic HMC-like identity (Fig. 3a, b). As a consequence, mice lacking *Hox5* genes show severe defects in respiratory function and perish at birth [50, 51]. Hox5 proteins act in conjunction with more MN-restricted fate determinants, including the POU-class homeodomain protein Scip (Pou3f1), which is also essential for respiratory function [52]. Downstream targets of Hox5 and Scip activities include genes encoding the cell adhesion proteins Cdh10 and Pcdh10, which appear to be important for PMC neurons to cluster into columnar groups [53].

Whether MMC neurons targeting specific epaxial muscles show the same degree of molecular diversity as HMC-derived MNs is less clear. While all MMC neurons can be defined by the maintenance of *Lhx3/4* expression, the specific determinants of MMC subtype-specific properties are poorly defined. A recent study investigating the function of Pbx transcription factors in spinal MN differentiation identified a novel repertoire of genes selectively expressed in mature MMC neurons [54]. Pbx proteins are known to be important cofactors for Hox proteins, and are essential for the specification of segmentally-restricted neuronal subtypes [55]. Mutation of *Pbx* genes in spinal MNs disrupts the specification of all Hox-dependent subtypes, with the majority of the remaining MNs consisting of MMC and HMC neurons. Surprisingly, removal of *Pbx* genes also leads a loss of the somatotopic organization of the remaining Hox-independent MMC and HMC populations. In *Pbx* mutants, MNs with MMC and HMC molecular identities are generated at all rostrocaudal spinal levels, but MNs of each type are randomly distributed within the ventral cord (Fig. 3c).

Loss of *Pbx* genes does not affect the ability of MMC and HMC neurons to select appropriate muscle targets [54], suggesting a specific function of Pbx targets in governing MN columnar organization. Gene targets acting downstream of Pbx proteins are therefore essential for the ability of axial MNs to coalesce into specific columnar groups. Identification of genes differentially expressed between normal and *Pbx* mutant MNs uncovered a novel repertoire of targets that are selectively expressed in MMC neurons (Fig. 3d). These downstream targets include the transcription factor *Mecom* (*MDS1/Evi1*), which marks postmitotic axial MNs and can be induced by forced misexpression of Lhx3 in non-MMC populations. The disorganization of axial MNs in *Pbx* mutants therefore appears to be a consequence of the disruption of regulatory programs acting in MMC neurons.

## Development of locomotor axial motor circuits in fish

While the connections established between axial MNs and muscle play important roles in shaping motor functions, how the activities of different classes of MNs are controlled during specific motor behaviors are less well-understood. Activation of specific MN subtypes is orchestrated through the inputs they receive from higher order “premotor” microcircuits within the spinal cord and brain. In many cases, these premotor networks assemble into rhythmically active central pattern generator (CPG) networks to control basic behaviors such as walking, swimming, and breathing [1, 56, 57]. Much of our understanding of the functional and electrophysiological properties of CPG networks stem from studies of axial muscle-driven motor circuits in the lamprey, which defined the core neuronal constituents of CPGs [58]. Recent studies in genetically tractable systems, such as zebrafish, have drawn new attention to the role of axial MNs in shaping functional properties of locomotor CPG networks.

The first movements of the embryonic zebrafish begin at 17 hpf with altering coil contractions of the trunk that increase in frequency until 19 hpf and decrease until 27 hpf [32]. These early spontaneous coiling contractions in the embryo are not dependent on synaptic transmission, but involve electrically coupled networks of a subset of premotor interneurons that are rhythmically active and dependent on gap junctions [33]. Ipsilateral neurons are electrically coupled and active simultaneously, while contralateral neurons are alternatively active [33]. At 21 hpf, zebrafish will partially coil in response to touch and, at 27 hpf, zebrafish will swim in response to touch. These touch responses, and swimming thereafter, depend on glutamaterigic and glycinergic chemical synaptic drive and descending inputs from the hindbrain [32, 33]. Propulsion during swimming is generated by alternating, neural-mediated waves of muscle contractions along the trunk of the fish.

The organization of MNs in the zebrafish spinal cord correlates with their functional role. This relationship is because the MNs are grouped according to which type of muscle fiber they innervate (Fig. 1d) [14]. For example, the dorsal most MNs innervate fast muscle and are involved in large, fast swimming. During swimming, MNs are recruited from slow to intermediate to fast, and, therefore, from ventral MNs to dorsal MNs. Target muscle is not the only defining factor between these groups of neurons, as firing pattern, input resistance, reliability, and oscillatory drive, are just a few of the intrinsic properties suspected to contribute to their differential recruitment [14, 59, 60].

Primary MNs, which innervate fast muscle, are known to be responsible for the initial spontaneous coiling contractions and later escape behavior in zebrafish, while various subsets of secondary MNs are necessary for all swim speeds. In a *ned1* mutant where secondary MNs degenerate, but primary MNs are preserved, normal spontaneous coiling contractions are present, but the fish cannot swim [33]. Although the purpose of these separate waves of neuronal birth remains elusive, some hypothesize primary MNs are necessary to form a base for the development of locomotor CPG in the early embryonic spinal cord [19].

Excitatory inputs onto axial MNs in zebrafish are provided by V2a interneurons defined by expression of the Chx10 transcription factor [61–63]. It has been shown that distinct V2a populations drive dorsal and ventral trunk musculature in zebrafish [60, 64, 65]. Studies in both zebrafish and lamprey disprove the previous notion that only left-right alternation CPGs existed in primitive axial muscle control [64, 66]. This differential input contributes to the non-synchronous activation of these muscle groups important for behaviors such as postural control. Independent control of dorsal and ventral ipsilateral muscles is suggested to have been a template for separate control of muscles on the same side of the body, such as those in limbs [67].

Zebrafish are able to modulate their swimming speed through the recruitment of distinct MN subtypes. While the MNs that drive different swimming speeds vary in anatomical size and excitability, studies suggest differential recruitment of neurons along the dorso-ventral axis is not dependent solely on intrinsic properties but also on preferential excitatory drive [67]. Analogous to zebrafish spinal MNs, interneurons are organized on the dorsal-ventral axis based on recruitment during swimming and birth order [62]. Dorsally positioned, early born V2a neurons are active during higher frequency swimming when ventral, late born V2a neurons are inhibited. At least for V2a neurons, the relation between position and recruitment order does not persist into adult stages [14, 61, 68, 69]. However, experiments in adult zebrafish have revealed preferential connections and reliable monosynaptic input from V2a neurons to proximal MNs recruited at the same frequency of swimming, consistent with the idea that different V2a neurons govern different speeds of locomotion [15, 61, 65, 69].

While premotor inputs have a significant influence on locomotor behavior, MNs are the ultimate gate to undulation in the zebrafish. Increasing evidence suggest MNs serve in an instructive manner to control the output of locomotor circuits. A recent study demonstrated that, in addition to having chemical synapses, some V2a interneurons in zebrafish are also electrically coupled to MNs via gap junctions. This coupling permits the backward propagation of electrical signals from MNs influencing the synaptic transmission and firing threshold of V2a interneurons, and therefore their recruitment during locomotion [70]. These gap junctions allow the MNs to control locomotor circuit function in a retrograde manner, causing the V2a interneurons and the MNs to act as a unit, which may contribute to the maintenance of locomotor rhythm generation.

## Functional diversity of axial motor circuits in tetrapods

While a primary function of axial MNs is to drive locomotion in zebrafish, in tetrapods MMC and HMC neurons play essential roles in multiple non-locomotor functions including breathing and maintaining spinal alignment. Some features of the locomotor CPG in fish appear to have been preserved in tetrapods to assist in limb-based locomotion. For example, in amphibian and reptile species undulation of spinal segments can be used to facilitate movements of limbs [71]. In mammals, particularly in bipedal species, axial MNs appear to have been largely dissociated from locomotor CPG networks, which likely played an important role in enabling new types of axial muscle-driven motor behaviors.

**Fig. 4:**
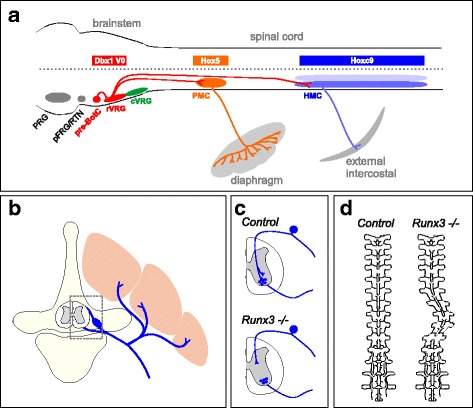
Diverse function of axial motor circuits in tetrapods. **a** Simplified diagram of respiratory networks for inspirational breathing. Rhythm generation in the preBötzinger (preBötz) complex is relayed to rostral ventral respiratory group (rVRG) neurons. rVRG neurons target PMC neurons and HMC neurons in the spinal cord. Connections between preBötz and rVRG neuron relies on *Dbx1* gene function. **b**-**d** Role of axial motor circuits in spinal alignment. **b** Axial muscles and nerves associated with vertebrae. Box indicates region magnified in panel **c**. **c** Consequences of *Runx3* mutation on the projection of proprioceptive sensory neurons in the spinal cord. Loss of *Runx3* leads to a loss of projections to MNs, and likely other classes of spinal interneurons. **d** Effect of *Runx3* mutation on vertebral alignment in adult mice

An important step in the evolution of axial motor circuits in tetrapods was the utilization of hypaxial muscle and its derivatives to support breathing on land. Expansion and contraction of the lungs during respiration is mediated by PMC and HMC neurons, which control the diaphragm and body wall muscle, respectively. In mammals, PMC and HMC firing is governed by CPG circuits located in the brainstem. Neurons in the preBötzinger (preBötz) complex and parafacial group provide the predominant rhythmic drive to PMC and HMC neurons during inspiratory and expiratory breathing [57]. Brainstem CPG networks target neurons in the ventral respiratory group (VRG) that in turn project to hypaxial and phrenic MNs within the spinal cord (Fig. 4a**)**. While the developmental logic that determines connectivity between preBötz, VRG, and spinal MNs is not fully understood, a recent study has shown that connectivity between preBötz and VRG neurons rely on a common transcription factor, Dbx1 [72]. Expression of Dbx1 is absent from MNs, suggesting other intrinsic factors are involved in establishing connectivity between VRG and axial MNs. Connections between brainstem respiratory centers and spinal MNs could rely on actions of segmentally-restricted fate determinants, such *Hox* genes, which differentiate PMC and HMC from other spinal MN subtypes (Fig. 4a) [73].

While motor circuits controlling breathing and locomotion rely on rhythmically active neural circuits, the development of motor circuits controlling postural stabilization and spinal alignment have been more difficult to study in mammals. In upright-walking bipedal vertebrates, the spine is kept in a relatively rigid configuration. Studies in humans indicate that coactivation of extensor and flexor axial muscles are essential for the load-bearing capacity and stability of the spine [74, 75]. The circuits that stabilize spinal alignment are not well characterized, but presumably require axial neural control systems that are fundamentally distinct from those controlling respiration in tetrapods and locomotion in fish.

A recent study in mice has provided evidence that sensory neurons play important roles in maintaining alignment of the spine. Mutation in the *Runx3* transcription factor, which is required for the development of muscle proprioceptive sensory neurons (pSNs) [76], leads to a progressive scoliosis of the spine (Fig. 4b-d) [77]. This phenotype does not appear to be a consequence of a requirement for *Runx3* function in other tissues, since similar results were observed after *Runx3* deletion specifically from pSNs. Although how this mutation affects the circuits involved in spinal stabilization is unclear, it is likely due to altered connections between pSNs and the axial motor circuits essential for maintaining posture. Loss and gain of function studies have shown that *Runx3* is required for the ability of pSNs to establish connections with MNs and other neural classes [77–79], suggesting that the *Runx3* mutant phenotype is due to the disruption of local sensory-motor spinal reflex circuits. In addition, mutations that affect the function of the MMC-restricted transcription factor *Mecom* also causes abnormal bending of the spine [80], raising the possibility that this phenotype is also consequence of altered connectivity between axial MNs and premotor neural populations.

## Developmental mechanisms of axial motor circuit assembly in tetrapods

**Fig. 5:**
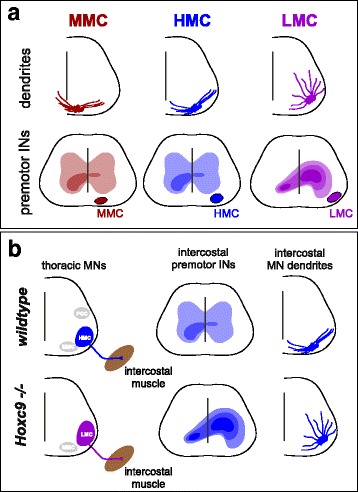
Developmental mechanisms of axial motor circuit assembly. **a** Dendritic morphology and premotor input pattern for MN columnar subtypes. MMC neurons have dendrites that extend across the midline and their monosynaptic premotor inputs are distributed across both sides of the spinal cord. Like MMC neurons, HMC neuron dendrites extend medio-laterally and have a similar premotor input distribution pattern. LMC neurons have radially organized dendrites and receive premotor inputs predominantly from ipsilateral spinal interneurons. Darker shading indicates higher density of interneurons connected to MNs. **b** Effect of *Hoxc9* mutation on premotor input pattern. In *Hoxc9* mutants, thoracic HMC neurons are converted to LMC fate, while MMC neurons are grossly unaffected. In *Hoxc9* mutants, ectopic LMC neurons still project to intercostal muscle. The dendritic pattern of thoracic MNs in *Hoxc9* mutants becomes more limb-like, and MNs projecting to intercostal muscle receive a higher distribution of inputs from ipsilateral premotor interneurons. Diagram based on data in [84]

The distinct use of MMC neurons in locomotion and posture, while HMC and HMC-like MNs are essential for breathing, raise the question of how premotor circuits dedicated to specific motor functions target the appropriate axial MN subtype. While the answer to this question is largely unknown, studies characterizing the distribution of spinal interneurons connected to specific MN columnar subtypes have provided a partial answer. Rabies-based monosynaptic tracing of interneurons connected to MMC and HMC neurons revealed that axial MNs receive local spinal premotor inputs that are evenly distributed across both sides of the spinal cord (Fig. 5a). In contrast, limb MNs receive inputs predominantly from premotor interneurons on the ipsilateral side of the spinal cord [81]. Axial MN dendritic arborization patterns are also distinct from those of limb MNs, which may help determine their specific connectivity with premotor interneuron populations (Fig. 5a). MMC neurons have dendrites that extend across the midline, which appears to enable them to capture a greater proportion of inputs from contralateral interneuron populations, and establish connectivity with interneurons distinct from those of HMC neurons. In contrast, limb-innervating LMC neurons are found in more lateral and dorsal regions of the spinal cord and have radially-projecting dendrites, which may afford them greater input from ipsilateral interneuron populations.

Do the molecular identities and/or positional differences between MN subtypes determine their premotor input pattern and function? The ability to genetically alter the composition of MN subtypes within the mouse spinal cord provides evidence that MN subtype identity plays an important role in determining the functional properties of spinal circuits. Conversion of limb MNs to an axial HMC fate, through deletion of limb MN determinant *Foxp1*, leads to the loss of limb-specific motor output patterns [82, 83]. In the absence of *Foxp1,* the normal alternation of limb-flexor and -extensor firing patterns is lost, and the remaining HMC-like populations fire in a predominantly flexor-like pattern.

Recent studies also indicate that determinants of MN columnar identity play crucial roles in defining the patterns and types of synaptic inputs that MNs receive [84]. Transformation of thoracic HMC neurons to a limb-level LMC fate, through mutation of the *Hoxc9* gene [85], shifts spinal premotor inputs to predominantly ipsilateral populations (Fig. 5b). In *Hoxc9* mutants, the transformed HMC populations also settle in a more dorsolateral position, and their dendrites project radially, similar to those of limb-innervating MNs (Fig. 5b) [84]. While these studies do not resolve the basic question of how differences between HMC and MMC inputs are achieved, they suggest that intrinsic differences between MN molecular identity, dendritic morphology, and position contribute to shaping the pattern of connection within the motor circuits. How these genetic manipulations affect the function of axial motor circuits remains to be determined. Nevertheless, analyses of *Foxp1* and *Hoxc9* mutants indicate that the columnar identity of spinal MNs plays a significant role in determining the architecture and output patterns of spinal circuits.

## Conclusions

Studies on the development of neural circuits controlling axial muscles have provided valuable insights into how specific motor functions develop and have evolved in the vertebrate lineage. While we have a fairly in depth understanding of the genetic programs controlling the specification of tetrapod axial MN subtypes, how these functionally diverse populations are connected to appropriate higher order circuits remains to be determined. Recent studies showing that MN-intrinsic programs contribute to differences in the patterns of premotor connectivity between limb and axial MNs suggests a general mechanism through which motor circuits are assembled, as a function of molecular differences in their target MN populations. Further functional studies on the consequences of disrupting MN differentiation could provide a means to test the role of MN subtype identity in the development of axial circuits essential for breathing and spinal alignment.

Comparisons between species that use axial MNs for distinct functions have provided insights into how different motor behaviors are specified during development. Although this review has focused on vertebrate development, many of the intrinsic molecular features of axial MNs appear to be conserved in invertebrates. Similar to vertebrates, in *Drosophila* and *C. elegans* subtypes of MNs can be defined by expression of the transcription factors, Hb9, Lhx3, and Isl1/2 [86]. Since it is thought the ancestor to all bilaterians had a fairly complex nervous system [87, 88], and likely used an axial-like locomotor circuit to move, it would be informative to know the extent to which the neural circuits governing axial muscle-driven locomotion have been preserved across animal species.

If an axial locomotor circuit represents the ancestral condition in the common ancestor to bilaterians, then what mechanisms were employed to generate the distinct neural circuits present in mammals? One example of how motor circuits have changed is the use of axial muscle for locomotion in fish, versus their non-locomotor functions in tetrapods. Whether these differences reflect whole-sale changes in spinal circuits, or changes in a limited number of circuit components remains to be determined. Further cross-species comparisons of the functional roles of specific interneuron and motor neuron subtypes will likely provide important clues into how axial motor circuits are established during development and have evolved across the animal kingdom.

## Abbreviations

CaP: Caudal primary motor neuron
CPG: Central pattern generator
dpf: Days post fertilization
dRoP: Dorsal rostral primary motor neuron
ei: External intercostal muscle
eo: External oblique muscle
ESC: Embryonic stem cell
FGF: Fibroblast growth factor
FGFR1: Fibroblast growth factor receptor 1
HMC: Hypaxial motor column
hpf: Hours post fertilization
ii: Internal intercostal muscle
ilio: Iliocostalis muscle
lc: Levator costae muscle
LMC: Lateral motor column
long: Longissimus muscle
MiP: Middle primary motor neuron
MMC: Medial motor column
MN: Motor neuron
PGC: Preganglionic motor column
PMC: Phrenic motor column
pMN: Motor neuron progenitor
pSN: Proprioceptive sensory neuron
sc: Subcostalis muscle
Shh: Sonic hedgehog
sr: Caudal serratus muscle
tv: Transversospinalis muscle
VRG: Ventral respiratory group
vRoP: Ventral rostral primary motor neuron

## References

[1] Kiehn O (2016). Decoding the organization of spinal circuits that control locomotion. Nat Rev Neurosci.

[2] Catela C, Shin MM, Dasen JS (2015). Assembly and function of spinal circuits for motor control. Annu Rev Cell Dev Biol.

[3] Fetcho JR (1987). A review of the organization and evolution of motoneurons innervating the axial musculature of vertebrates. Brain Res.

[4] Romer AS, Parsons TS (1977). The vertebrate body.

[5] Romanes GJ (1964). The motor pools of the spinal cord. Prog Brain Res.

[6] Romanes GJ (1951). The motor cell columns of the lumbo-sacral spinal cord of the cat. J Comp Neurol.

[7] Landmesser L (1978). The distribution of motoneurones supplying chick hind limb muscles. J Physiol.

[8] Hollyday M, Jacobson RD (1990). Location of motor pools innervating chick wing. J Comp Neurol.

[9] Demireva EY, Shapiro LS, Jessell TM, Zampieri N (2011). Motor neuron position and topographic order imposed by beta- and gamma-catenin activities. Cell.

[10] Price SR, De Marco Garcia NV, Ranscht B, Jessell TM (2002). Regulation of motor neuron pool sorting by differential expression of type II cadherins. Cell.

[11] Tsuchida T, Ensini M, Morton SB, Baldassare M, Edlund T, Jessell TM, Pfaff SL (1994). Topographic organization of embryonic motor neurons defined by expression of LIM homeobox genes. Cell.

[12] Gutman CR, Ajmera MK, Hollyday M (1993). Organization of motor pools supplying axial muscles in the chicken. Brain Res.

[13] Smith CL, Hollyday M (1983). The development and postnatal organization of motor nuclei in the rat thoracic spinal cord. J Comp Neurol.

[14] Ampatzis K, Song J, Ausborn J, El Manira A (2013). Pattern of innervation and recruitment of different classes of motoneurons in adult zebrafish. J Neurosci.

[15] McLean DL, Fan J, Higashijima S, Hale ME, Fetcho JR (2007). A topographic map of recruitment in spinal cord. Nature.

[16] Liu DW, Westerfield M (1988). Function of identified Motoneurones and coordination of primary and secondary motor systems during Zebra fish swimming. J Physiol.

[17] Jung H, Baek M, D'Elia KP, Boisvert C, Currie PD, Tay BH, Venkatesh B, Brown SM, Heguy A, Schoppik D, Dasen JS (2018). The ancient origins of neural substrates for land walking. Cell.

[18] Stifani N (2014). Motor neurons and the generation of spinal motor neuron diversity. Front Cell Neurosci.

[19] Dessaud E, McMahon AP, Briscoe J (2008). Pattern formation in the vertebrate neural tube: a sonic hedgehog morphogen-regulated transcriptional network. Development.

[20] Jessell TM (2000). Neuronal specification in the spinal cord: inductive signals and transcriptional codes. Nat Rev Genet.

[21] Dasen JS, De Camilli A, Wang B, Tucker PW, Jessell TM (2008). Hox repertoires for motor neuron diversity and connectivity gated by a single accessory factor, FoxP1. Cell.

[22] Sharma K, Sheng HZ, Lettieri K, Li H, Karavanov A, Potter S, Westphal H, Pfaff SL (1998). LIM homeodomain factors Lhx3 and Lhx4 assign subtype identities for motor neurons. Cell.

[23] Sharma K, Leonard AE, Lettieri K, Pfaff SL (2000). Genetic and epigenetic mechanisms contribute to motor neuron pathfinding. Nature.

[24] Dasen JS, Jessell TM (2009). Hox networks and the origins of motor neuron diversity. Curr Top Dev Biol.

[25] Soundararajan P, Miles GB, Rubin LL, Brownstone RM, Rafuse VF (2006). Motoneurons derived from embryonic stem cells express transcription factors and develop phenotypes characteristic of medial motor column neurons. J Neurosci.

[26] Peljto M, Dasen JS, Mazzoni EO, Jessell TM, Wichterle H (2010). Functional diversity of ESC-derived motor neuron subtypes revealed through intraspinal transplantation. Cell Stem Cell.

[27] Agalliu D, Takada S, Agalliu I, McMahon AP, Jessell TM (2009). Motor neurons with axial muscle projections specified by Wnt4/5 signaling. Neuron.

[28] Tan GC, Mazzoni EO, Wichterle H (2016). Iterative role of notch signaling in spinal motor neuron diversification. Cell Rep.

[29] Tosney KW (1988). Proximal tissues and patterned neurite outgrowth at the lumbosacral level of the Chick-embryo - partial and complete deletion of the somite. Dev Biol.

[30] Tosney KW (1987). Proximal tissues and patterned neurite outgrowth at the lumbosacral level of the Chick-embryo - deletion of the Dermamyotome. Dev Biol.

[31] Shirasaki R, Lewcock JW, Lettieri K, Pfaff SL (2006). FGF as a target-derived chemoattractant for developing motor axons genetically programmed by the LIM code. Neuron.

[32] Saint-Amant L, Drapeau P (1998). Time course of the development of motor behaviors in the zebrafish embryo. J Neurobiol.

[33] Drapeau P, Saint-Amant L, Buss RR, Chong M, McDearmid JR, Brustein E (2002). Development of the locomotor network in zebrafish. Prog Neurobiol.

[34] Moreno RL, Ribera AB (2009). Zebrafish motor neuron subtypes differ electrically prior to axonal outgrowth. J Neurophysiol.

[35] Myers PZ, Eisen JS, Westerfield M (1986). Development and axonal outgrowth of identified motoneurons in the zebrafish. J Neurosci.

[36] Lewis KE, Eisen JS. From cells to circuits: development of the zebrafish spinal cord. Prog Neurobiol. 2003;69:419–49. 10.1016/s0301-0082(03)00052-2 12880634

[37] Appel B, Korzh V, Glasgow E, Thor S, Edlund T, Dawid IB, Eisen JS (1995). Motoneuron fate specification revealed by patterned LIM homeobox gene expression in embryonic zebrafish. Development.

[38] Seredick SD, Van Ryswyk L, Hutchinson SA, Eisen JS. Zebrafish Mnx proteins specify one motoneuron subtype and suppress acquisition of interneuron characteristics. Neural Dev. 2012;7:35. 10.1186/1749-8104-7-35 PMC3570319 23122226

[39] Park HC, Mehta A, Richardson JS, Appel B (2002). olig2 is required for zebrafish primary motor neuron and oligodendrocyte development. Dev Biol.

[40] Hutchinson SA, Cheesman SE, Hale LA, Boone JQ, Eisen JS (2007). Nkx6 proteins specify one zebrafish primary motoneuron subtype by regulating late islet1 expression. Development.

[41] Seredick S, Hutchinson SA, Van Ryswyk L, Talbot JC, Eisen JS (2014). Lhx3 and Lhx4 suppress Kolmer-Agduhr interneuron characteristics within zebrafish axial motoneurons. Development.

[42] Hutchinson SA, Eisen JS (2006). Islet1 and Islet2 have equivalent abilities to promote motoneuron formation and to specify motoneuron subtype identity. Development.

[43] Jung H, Dasen JS (2015). Evolution of patterning systems and circuit elements for locomotion. Dev Cell.

[44] Beattie CE, Hatta K, Halpern ME, Liu HB, Eisen JS, Kimmel CB (1997). Temporal separation in the specification of primary and secondary motoneurons in zebrafish. Dev Biol.

[45] Menelaou E, McLean DL (2012). A gradient in endogenous rhythmicity and oscillatory drive matches recruitment order in an axial motor pool. J Neurosci.

[46] Rousso DL, Gaber ZB, Wellik D, Morrisey EE, Novitch BG (2008). Coordinated actions of the forkhead protein Foxp1 and Hox proteins in the columnar organization of spinal motor neurons. Neuron.

[47] Catela C, Shin MM, Lee DH, Liu JP, Dasen JS (2016). Hox proteins coordinate motor neuron differentiation and connectivity programs through ret/Gfr alpha genes. Cell Rep.

[48] Lacombe J, Hanley O, Jung H, Philippidou P, Surmeli G, Grinstein J, Dasen JS. Genetic and functional modularity of Hox activities in the specification of limb-innervating motor neurons. PLoS Genet. 2013;9:e1003184. 10.1371/journal.pgen.1003184 PMC3554521 23359544

[49] Mendelsohn AI, Dasen JS, Jessell TM (2017). Divergent Hox coding and evasion of retinoid signaling specifies motor neurons innervating digit muscles. Neuron.

[50] Philippidou P, Walsh CM, Aubin J, Jeannotte L, Dasen JS (2012). Sustained Hox5 gene activity is required for respiratory motor neuron development. Nat Neurosci.

[51] Landry-Truchon K, Fournier S, Houde N, Rousseau JP, Jeannotte L, Kinkead R (2017). Respiratory consequences of targeted losses of Hoxa5 gene function in mice. J Exp Biol.

[52] Bermingham JR, Scherer SS, O'Connell S, Arroyo E, Kalla KA, Powell FL, Rosenfeld MG (1996). Tst-1/Oct-6/SCIP regulates a unique step in peripheral myelination and is required for normal respiration. Genes Dev.

[53] Machado CB, Kanning KC, Kreis P, Stevenson D, Crossley M, Nowak M, Iacovino M, Kyba M, Chambers D, Blanc E, Lieberam I (2014). Reconstruction of phrenic neuron identity in embryonic stem cell-derived motor neurons. Development.

[54] Hanley O, Zewdu R, Cohen LJ, Jung H, Lacombe J, Philippidou P, Lee DH, Selleri L, Dasen JS (2016). Parallel Pbx-dependent pathways govern the coalescence and fate of motor columns. Neuron.

[55] Moens CB, Selleri L (2006). Hox cofactors in vertebrate development. Dev Biol.

[56] Grillner S, Jessell TM (2009). Measured motion: searching for simplicity in spinal locomotor networks. Curr Opin Neurobiol.

[57] Feldman JL, Del Negro CA, Gray PA (2013). Understanding the rhythm of breathing: so near, yet so far. Ann Rev Physiol.

[58] Grillner S (2006). Biological pattern generation: the cellular and computational logic of networks in motion. Neuron.

[59] Wang WC, Brehm P (2017). A gradient in synaptic strength and plasticity among Motoneurons provides a peripheral mechanism for locomotor control. Curr Biol.

[60] Menelaou E, VanDunk C, McLean DL (2014). Differences in the morphology of spinal V2a neurons reflect their recruitment order during swimming in larval zebrafish. J Comp Neurol.

[61] Eklof-Ljunggren E, Haupt S, Ausborn J, Dehnisch I, Uhlen P, Higashijima S, El Manira A (2012). Origin of excitation underlying locomotion in the spinal circuit of zebrafish. Proc Natl Acad Sci U S A.

[62] Kimura Y, Okamura Y, Higashijima S (2006). Alx, a zebrafish homolog of Chx10, marks ipsilateral descending excitatory interneurons that participate in the regulation of spinal locomotor circuits. J Neurosci.

[63] Ljunggren EE, Haupt S, Ausborn J, Ampatzis K, El Manira A (2014). Optogenetic activation of excitatory premotor interneurons is sufficient to generate coordinated locomotor activity in larval zebrafish. J Neurosci.

[64] Bagnall MW, McLean DL (2014). Modular organization of axial microcircuits in zebrafish. Science.

[65] McLean DL, Masino MA, Koh IY, Lindquist WB, Fetcho JR (2008). Continuous shifts in the active set of spinal interneurons during changes in locomotor speed. Nat Neurosci.

[66] Wallen P, Grillner S, Feldman JL, Bergelt S (1985). Dorsal and ventral myotome Motoneurons and their input during fictive locomotion in lamprey. J Neurosci.

[67] McLean DL, Dougherty KJ (2015). Peeling back the layers of locomotor control in the spinal cord. Curr Opin Neurobiol.

[68] Gabriel JP, Ausborn J, Ampatzis K, Mahmood R, Eklof-Ljunggren E, El Manira A (2011). Principles governing recruitment of motoneurons during swimming in zebrafish. Nat Neurosci.

[69] Ampatzis K, Song J, Ausborn J, El Manira A (2014). Separate microcircuit modules of distinct v2a interneurons and motoneurons control the speed of locomotion. Neuron.

[70] Song J, Ampatzis K, Bjornfors ER, El Manira A (2016). Motor neurons control locomotor circuit function retrogradely via gap junctions. Nature.

[71] Chevallier S, Ijspeert AJ, Ryczko D, Nagy F, Cabelguen JM (2008). Organisation of the spinal central. Pattern generators for locomotion in the salamander: biology and modelling. Brain Res Rev.

[72] Wu J, Capelli P, Bouvier J, Goulding M, Arber S, Fortin G (2017). A V0 core neuronal circuit for inspiration. Nat Commun.

[73] Philippidou P, Dasen JS (2013). Hox genes: choreographers in neural development, architects of circuit organization. Neuron.

[74] Crisco JJ, Panjabi MM, Yamamoto I, Oxland TR (1992). Euler stability of the human ligamentous lumbar spine. Part II: experiment. Clin Biomech (Bristol, Avon).

[75] Cholewicki J, Panjabi MM, Khachatryan A (1997). Stabilizing function of trunk flexor-extensor muscles around a neutral spine posture. Spine (Phila Pa 1976).

[76] Levanon D, Bettoun D, Harris-Cerruti C, Woolf E, Negreanu V, Eilam R, Bernstein Y, Goldenberg D, Xiao C, Fliegauf M, Kremer E, Otto F, Brenner O, Lev-Tov A, Groner Y (2002). The Runx3 transcription factor regulates development and survival of TrkC dorsal root ganglia neurons. EMBO J.

[77] Blecher R, Krief S, Galili T, Biton IE, Stern T, Assaraf E, Levanon D, Appel E, Anekstein Y, Agar G, Groner Y, Zelzer E (2017). The proprioceptive system masterminds spinal alignment: insight into the mechanism of scoliosis. Dev Cell.

[78] Chen AI, de Nooij JC, Jessell TM (2006). Graded activity of transcription factor Runx3 specifies the laminar termination pattern of sensory axons in the developing spinal cord. Neuron.

[79] Kramer I, Sigrist M, de Nooij JC, Taniuchi I, Jessell TM, Arber S (2006). A role for Runx transcription factor signaling in dorsal root ganglion sensory neuron diversification. Neuron.

[80] Juneja SC, Vonica A, Zeiss C, Lezon-Geyda K, Yatsula B, Sell DR, Monnier VM, Lin S, Ardito T, Eyre D, Reynolds D, Yao Z, Awad HA, Yu H, Wilson M, Honnons S, Boyce BF, Xing L, Zhang Y, Perkins AS (2014). Deletion of Mecom in mouse results in early-onset spinal deformity and osteopenia. Bone.

[81] Goetz C, Pivetta C, Arber S (2015). Distinct limb and trunk premotor circuits establish laterality in the spinal cord. Neuron.

[82] Machado TA, Pnevmatikakis E, Paninski L, Jessell TM, Miri A (2015). Primacy of flexor locomotor pattern revealed by ancestral reversion of motor neuron identity. Cell.

[83] Hinckley CA, Alaynick WA, Gallarda BW, Hayashi M, Hilde KL, Driscoll SP, Dekker JD, Tucker HO, Sharpee TO, Pfaff SL (2015). Spinal locomotor circuits develop using hierarchical rules based on Motorneuron position and identity. Neuron.

[84] Baek M, Pivetta C, Liu JP, Arber S, Dasen JS (2017). Columnar-intrinsic cues shape premotor input specificity in locomotor circuits. Cell Rep.

[85] Jung H, Lacombe J, Mazzoni EO, Liem KF, Grinstein J, Mahony S, Mukhopadhyay D, Gifford DK, Young RA, Anderson KV, Wichterle H, Dasen JS (2010). Global control of motor neuron topography mediated by the repressive actions of a single hox gene. Neuron.

[86] Thor S, Thomas JB (2002). Motor neuron specification in worms, flies and mice: conserved and 'lost' mechanisms. Curr Opin Genet Dev.

[87] De Robertis EM, Sasai Y (1996). A common plan for dorsoventral patterning in Bilateria. Nature.

[88] Arendt D, Denes AS, Jekely G, Tessmar-Raible K (2008). The evolution of nervous system centralization. Philos Trans R Soc Lond Ser B Biol Sci.

